# Large Basilar Aneurysm with Posterior Inferior Cerebellar Artery Stroke and Consequential Fatal Subarachnoid Hemorrhage

**DOI:** 10.1155/2012/204585

**Published:** 2012-09-17

**Authors:** Rohit Patel, Brandon Allen, Bobby Desai

**Affiliations:** Department of Emergency Medicine, University of Florida College of Medicine, 1329 SW 16th Street, P.O. Box 100186, Gainesville, FL 32610-0186, USA

## Abstract

Basilar artery aneurysm presenting a stroke is rare, and we present a case of this along with a discussion of the management options available.

## 1. Introduction

This is a case report of a patient with a large basilar aneurysm presenting to our facility. The patient presented with symptoms consistent with an ischemic stroke. On hospital day 3, the patient had an acute decompensation with further imaging confirming a fatal subarachnoid hemorrhage. The entity of large basilar artery aneurysm presenting as a stroke is exceedingly rare. The sequelae and prognostic ramifications of this condition make it important to examine and recognize.

## 2. Case Report 

A 64-year-old male presented to the emergency department as a “stroke alert” with new left-sided facial droop and aphasia one hour prior to arrival. The patient had a past medical history of hypertension, coronary artery disease, and ischemic cardiomyopathy with heart failure. Emergency Medical Services (EMS) stated that on their arrival to the scene, the patient had aphasia, left-sided facial droop and was somnolent. EMS reported that the patient had a Glascow Coma Score of 14, and the patient was easy to arouse to verbal stimuli. Pertinent positives in the review of systems included neck stiffness, nausea, vomiting, speech change, headache, and loss of balance. The patient's vital signs on arrival included blood pressure of 179/102 mm Hg, pulse rate of 79 beats per minute, temperature 36.9 degrees Celsius (oral), respiratory rate of 20 breaths per minute, and a pulse oximeter reading of 97%. Pertinent physical exam findings included an expressive aphasia, left-sided facial droop, nonfatiguable horizontal and vertical nystagmus bilaterally, full range-of-motion of the neck, no pronator drift bilaterally with normal strength in the upper and lower extremities bilaterally, past-pointing of the left upper extremity on finger-to-nose examination, and two plus pitting edema of the lower extremities bilaterally.

**Figure 1:**
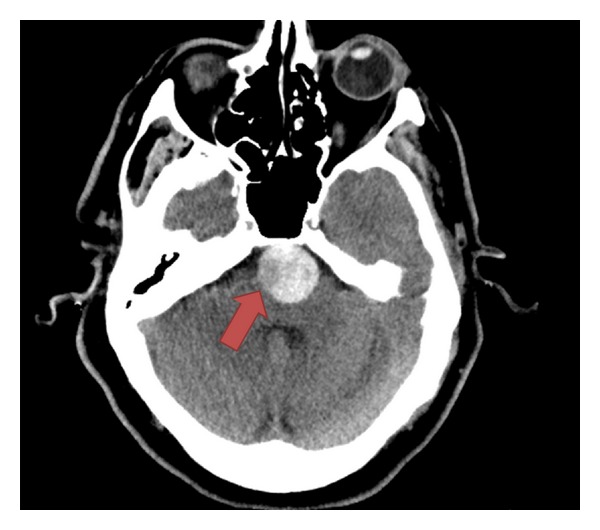
CT head without contrast: 25 mm basilar artery aneurysm with partial thrombosis and no evidence of rupture.

**Figure 2:**
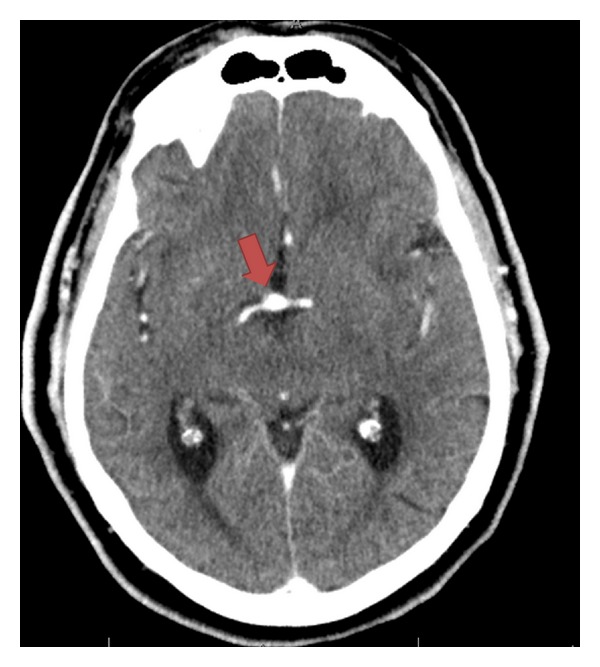
There is a second saccular-type aneurysm originating at the posterior cerebral artery segment of the left internal carotid artery. The width of the aneurysm fundus measures 8 mm; the aneurysm neck measures 2.7 mms; the neck-to-dome distance measures 8 mm. The long axis of the aneurysm projects inferomedially.

**Figure 3:**
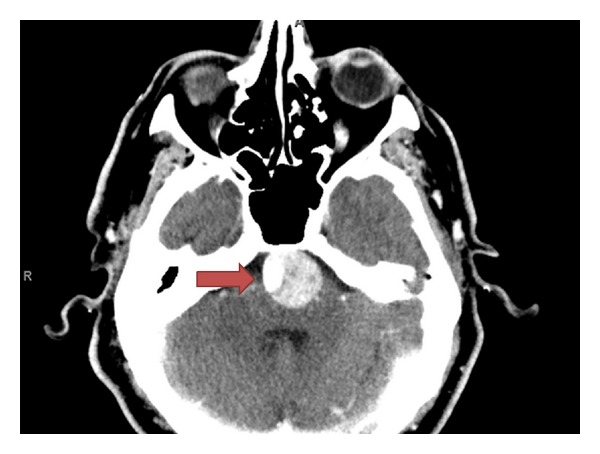
Head CTA: There is a very large aneurysm of the basilar artery measuring 25 mm in diameter, which shows a large amount of thrombus within it. The right lateral aspect of the thrombus remains canalized, extending into the basilar tip. There is associated mass effect on the medulla and pons. There is no evidence of aneurysm rupture.

**Figure 4:**
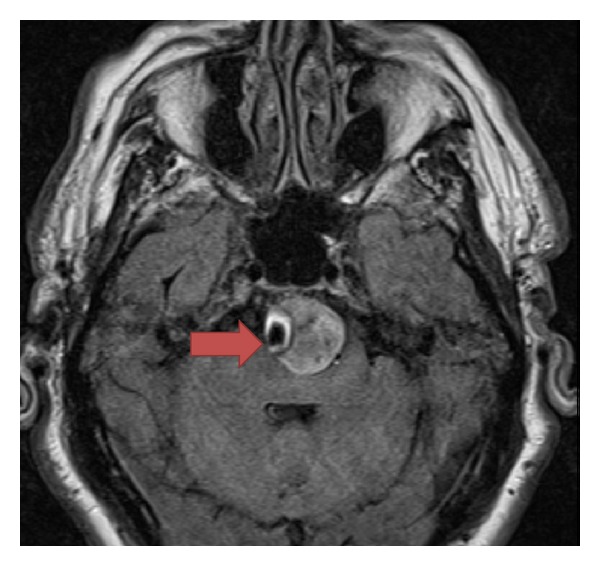
MRI Interpretation: Large partially thrombosed 25 mm basilar artery aneurysm without evidence of rupture. There is an acute or early subacute infarct within the left posterior inferior cerebellar artery distribution. There is also a questionable small focus of acute or early subacute infarct at the left posterolateral pontomedullary junction. There is no hydrocephalus or herniation.

The patient underwent a CT with angiography of the head and neck per the department's stroke protocol (see Figures 1, 2, and 3) which revealed a 25 mm basilar aneurysm with partial thrombosis and no evidence of rupture with a secondary saccular aneurysm of the left posterior cerebral artery. The neurosurgery and neurology services were both consulted with no direct intervention recommended. The patient was started on anticoagulation with heparin due to thrombus within the aneurysm and MRI performed (Figure 4). A radial arterial line was placed and the patient was kept normotensive prior to being admitted to the neurology intensive care unit without further decompensation prior to transport.

**Figure 5:**
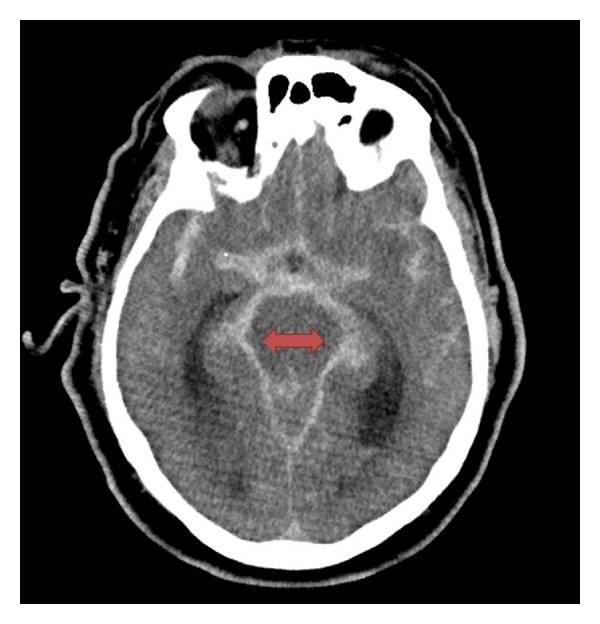
Hospital day 3 CT head without contrast: Diffuse subarachnoid blood products. There are also blood products layering within the occipital horns of the bilateral lateral ventricles. There are also subarachnoid blood products extending inferiorly surrounding the cervical cord. This is consistent with rupture of the patient's known basilar artery aneurysm. The ventricles are further enlarged with findings of transependymal CSF migration. There is no herniation.

On hospital day three, the patient became difficult to arouse and his heparin infusion was discontinued. An emergent noncontrast CT of the head (Figure 5) revealed diffuse blood products in the subarachnoid spaces and early ventricular dilation. The patient was reevaluated after returning from CT and found to have fixed, midposition pupils, absent corneal reflexes, and no response to painful stimuli. The patient was made Do Not Resuscitate (DNR) due to his terminal condition and he subsequently expired.

## 3. Discussion

Basilar artery aneurysms with subsequent thrombosis can be very difficult to manage in the critical care setting. The difficulty lies within the acute management of ischemic events in patients with large basilar artery aneurysms due to the involvement of both bleeding and thrombosis. Our patient presented with ischemic symptoms with a very large basilar artery aneurysm, and therefore posed difficulties of treatment for ischemia versus aneurysm rupture prevention. Management concerns include hemodynamic optimization, identification of etiology and rate of progression, risk of rupture, and neurosurgical consultation/intervention. 

### 3.1. Hemodynamic Optimization

Blood pressure management varies significantly for ischemic stroke versus intracranial bleeding from aneurysm. The risk to the penumbra area of the ischemic stroke must be weighed against the risk of rupture due to increased pressure on the aneurysm. One study showed that nonruptured aneurysms tend to have diffuse inflow jets, large areas of flow impaction, and simple stable flow patterns; in contrast to ruptured aneurysms which tend to have concentrated inflow jets, small flow impingement regions, and complex, unstable flow patterns [1]. A separate study supports that hemodynamic characteristics derived from image-based computational models can be used to identify cerebral aneurysms at high risk for rupture. The authors found an aneurysm that had concentrated inflow jet that impacted onto a small region of the dome of the aneurysm and created a complex, unstable flow pattern [2].

### 3.2. Etiology and Rate of Progression

Fusiform cerebral aneurysms can be divided into acute dissecting aneurysms and chronic fusiform or dolichoectatic aneurysms, which slowly enlarge and can be asymptomatic or associated with cranial nerve dysfunction, ischemic stroke, or subarachnoid hemorrhage [3, 4]. This distinction is often difficult even with advanced neuroimaging techniques. More than half of large cerebral aneurysms with a diameter of 20 mm or more are known to have thrombi [5]. The question arises is whether treatment should ensue for brain ischemia. Anticoagulation therapy at conventional doses seems to increase the risk of intracranial bleeding, but the role of further antiplatelet agents is less clear [6, 7].

### 3.3. Risk of Rupture

The mechanisms for initiation, progression, and rupture of intracranial aneurysms are not well understood. The International Study of Unruptured Intracranial Aneurysms (ISUIA) provided compelling evidence that natural history is different for patients with history of SAH due to a separate aneurysm [8]. In a recent review, it was confirmed that size of the aneurysm, posterior circulation aneurysms, and a history of subarachnoid hemorrhage (SAH) were independent predictors for subsequent SAH [9]. It was found in this series that aneurysms larger than 10 mm in diameter demonstrated a higher probability of rupture than the aneurysm in the range of 0 to 4.9 mm diameter). Also in this series it was noted that risk of rupture in posterior circulation aneurysms was significantly higher than that of anterior circulation aneurysms. Genetic analysis has shown that Japanese and/or Finnish descendants have a higher potential for rupture [10]. 

### 3.4. Neurosurgical Interventions

Therapy for aneurysms of the basilar artery is difficult due to the presence of perforating vessels, the proximity to the brainstem and the exiting cranial nerves, in conjunction with the surgical approaches necessary to access the basilar artery [11]. Although surgical clipping and endovascular coiling are the most common treatments, they continue to result in high rates of failure and complications [12, 13]. Coil embolization of the large basilar apex aneurysms is not optimal due to high rate of recurrence which can be as high as 41% at 6-to 12-month followup [14, 15]. A recent report has shown success with surgical clip occlusion of the proximal basilar artery offering a high rate of angiographic cure in a single procedure for patients with complex basilar artery aneurysms [16].
